# Stick to It, Brethren

**Published:** 1889-03

**Authors:** 


### STICK TO IT, BRETHREN, EVEN THOUGH YOU KNOW YOU'RE WRONG.

There was a deadlock between the American Board of Commissioners of Foreign Missions and the Berkley Street Church relative to the ordination of the Rev. William H. Noyes for missionary work. The Board refuses to appoint Mr. Noyes because he declines to withdraw the statements made by him to the committee at the time of his previous application for appointment, which favor the hypothesis of a probation after death—this hypothesis being, as he stated, "in harmony with Scripture,"

and one which "honors Christ in giving completeness to his work," and which is to him "a necessary corollary to belief in the universality of the atonement." In their decision the Prudential Committee say, in closing :

"We must express the strong conviction that should he be sent out as an independent missionary it would be highly inexpedient, in view of all the circumstances, that he be sent to any missionary field under the care of the American Board, since such a course would be, in our view, almost inevitably divisible in its results, both at home and abroad."

This action of the Board does not meet the approval of the leading orthodox preachers here.

